# Comparative immune responses against *Psoroptes ovis* in two cattle breeds with different susceptibility to mange

**DOI:** 10.1186/s13567-015-0277-x

**Published:** 2015-11-19

**Authors:** Charlotte Sarre, Ana González-Hernández, Stefanie Van Coppernolle, Rika Grit, Korneel Grauwet, Frederik Van Meulder, Koen Chiers, Wim Van den Broeck, Peter Geldhof, Edwin Claerebout

**Affiliations:** Department of Virology, Parasitology and Immunology, Faculty of Veterinary Medicine, Ghent University, Merelbeke, Belgium; Department of Pathology, Bacteriology and Poultry Diseases, Faculty of Veterinary Medicine, Ghent University, Merelbeke, Belgium; Department of Morphology, Faculty of Veterinary Medicine, Ghent University, Merelbeke, Belgium

## Abstract

The sheep scab mite, *Psoroptes ovis*, is a major problem in the beef cattle industry, especially in Belgian Blue (BB) cattle. This breed is naturally more predisposed to psoroptic mange but reasons for this high susceptibility remain unknown. Different immune responses could be a potential cause; thus in this study, the cutaneous immune response and in vitro cellular immune response after antigen re-stimulation were examined in naturally infested BB. Cytokine production in the skin and in circulating re-stimulated peripheral blood mononuclear cells (PBMC) demonstrated a mixed pro-inflammatory Th2/Th17 profile, with transcription of IL-4, IL-13, IL-6 and IL-17. Strong IL-17 up-regulation in the skin of BB was associated with an influx of eosinophils and other immune cells, potentially leading towards more severe symptoms. Virtually no changes in cutaneous IFN-γ transcription were detected, while there was substantial IFN-γ up-regulation in re-stimulated PBMC from infested and uninfested animals, potentially indicating a role of this pro-inflammatory cytokine in the innate immune response. In Holstein–Friesian (HF) cattle, generally more resistant to *P. ovis* infection, a largely similar immunologic response was observed. Differences between HF and BB were the lack of cutaneous IL-17 response in infested HF and low transcription levels of IFN-γ and high IL-10 transcription in re-stimulated PBMC from both infested and uninfested animals. Further research is needed to identify potential cell sources and biological functions for these cytokines and to fully unravel the basis of this different breed susceptibility to *P. ovis*.

## Introduction

*Psoroptes ovis* (*P. ovis*), the causative agent of psoroptic mange, causes severe allergic dermatitis and intense pruritus in sheep and cattle. The disease is highly contagious and causes impaired animal welfare and major economic losses due to performance loss and substantial treatment costs in livestock production all over the world [1–3]. Compared to sheep, *P. ovis* in cattle has a more limited geographical distribution, with a hotspot in Belgium where a farm prevalence of 75% has been observed on Belgian Blue (BB) beef farms [4]. This cattle breed appears to be highly susceptible to the infection, whilst other beef breeds and dairy cattle, such as Holstein–Friesians (HF), seem to be more resistant, although the reason for this difference has not been clarified yet [5]. Host factors, such as genetic and immunologic variations, could be the cause of different breed susceptibility.

Previous research suggested that immune-depression was not responsible for higher susceptibility of BB, since BB animals developed *P. ovis* specific antibodies after infection, which correlated with the mite population density and the progression of the lesions. Moreover, in vitro culture of peripheral blood mononuclear cells (PBMC) showed an increased reactivity to mitogens [5–8]. In general, microscopic examination of the skin from infested ruminants reveals an increased dermal thickness and a superficial, perivascular dermatitis characterised by a fast influx of lymphocytes (including γδ T-cells in sheep), macrophages, mast cells, neutrophils and eosinophils [9–12]. Previous work on sheep scab demonstrated an up-regulation of genes encoding for pro-inflammatory and pro-allergic mediators [interleukin (IL)-1, IL-8, IL-6] and molecules involved in extravasation of immune cells [13, 14]. Several infectious diseases, including scabies, have been linked to CD4+ T-helper cell (Th) differentiation into specific Th1, Th2, Th17 or regulatory (Treg) lineages [15] and the pro-inflammatory response in the skin of *P. ovis* infested sheep evolves towards a Th2 immune response within 24 h after infection [13, 14, 16]. A similar pattern has been described in *Sarcoptes scabiei* infested mice and dogs with transcription of Th2 like cytokine profiles in lymph node cells and PBMC respectively [17, 18]. In addition, the epidermal differentiation complex (EDC) genes *filaggrin, involucrin* and *loricrin* are significantly down-regulated in infested sheep skin, indicating an impaired skin barrier function [9, 13] and markers of skin barrier disruption can also be found in circulating PBMC [16]. Furthermore, the systemic immune response parallels the cutaneous Th2 response as locally produced IL-4 and IL-13 stimulate PBMC to up-regulate IL-4R transcription and chemokine (C–C motif) receptor 3 (CCR3), an eosinophil activator [16]. Remarkably, both BB and HF cattle showed an immediate hypersensitivity reaction 1 h after intradermal injection of *Psoroptes cuniculi* antigen, but only in BB animals a delayed hypersensitivity reaction after 72 h could be recorded [5, 7]. Inherent differences in skin physiology and body composition could be responsible for this altered skin reaction in BB [19, 20].

As described for ticks [21, 22], susceptibility of cattle to mite infestations can also vary between individual animals. Increased susceptibility in sheep seems to correlate with higher numbers of eosinophils in the skin, larger lesions [11] and potential mutations in crucial EDC components [9, 13]. Humans suffering from severe sarcoptic scabies, better known as crusted or Norwegian scabies, show a predominant immunoglobulin E (IgE)-driven Th2 response with high levels of IL-5 and IL-13, in contrast to patients with ordinary scabies, who mainly express the Th1 cytokines IL-2 and IFN-γ [23–25]. In mice and rabbits, a skewed Th2/Th1 response favouring the cell-mediated Th1 response is linked with lower antibody titres, also resulting in more resistant individuals [17, 26, 27]. Recent investigations demonstrate an important role for Th17 cytokines in humans suffering from chronic and/or allergic inflammatory diseases, such as crusted scabies and psoriasis [28]. Moreover, in pigs with sarcoptic scabies, the development of severe symptoms is linked to higher levels of cutaneous IL-17, IL-23 but also Th2 cytokines IL-4 and IL-13 [23, 24, 28].

In conclusion, several potential reasons for differences in susceptibility to mange between breeds and between individual animals have been suggested in various species, with predominant roles for Th2 and Th17 cytokines. Therefore, the main objective of this study was to examine the cutaneous and in vitro cellular immune responses to *P. ovis* infestation in the highly susceptible BB cattle breed. In addition, these responses were compared with those in more resistant HF cattle.

## Materials and methods

### Animals and tissue collection

Ethical approval to conduct this study was obtained from the Ethical Committee of the Faculty of Veterinary Medicine, Ghent University (ethical approval number EC 2013/130). In total 28 animals were sampled: 20 BB and 8 HF. All animals were females of approximately 1 year (BB) or 2–3 years (HF) old. The BB cattle were uninfested animals (*n* = 8) or naturally infested animals with severe clinical signs of mange (*n* = 12). The degree of infestation was quantified by calculating the percentage of infested body surface (clinical index, CI) for each animal based on the method of Guillot [29]. Heavily infested animals had a CI of ≥10% (range 12–45%) and uninfested animals had a CI of 0%. The HF were also either uninfested (*n* = 4) with 0% CI or naturally infested with *P. ovis* (*n* = 4) with a CI of ≥1% (range 1–8%). Skin scrapings of all infested animals demonstrated a pure *P. ovis* infection. The absence of mites in the uninfested groups was confirmed by indirect examination (centrifugation-flotation after 10% potassium hydroxide digestion) of skin scrapings.

After confirmation of the presence or absence of *P. ovis* mites, two skin biopsies were taken per animal after shaving an area next to the tail base at the transition between healthy and infested skin, using a 4 mm diameter punch biopsy tool (Farla Medical). One biopsy was snap-frozen in liquid nitrogen and stored at −70 °C to allow RNA extraction. The second skin sample was stored in 4% formaldehyde and paraffin-embedded for histology and immunohistochemistry. In addition, blood was drawn from the *vena jugularis* using heparin-coated tubes and PBMC were isolated using a Lymphoprep density gradient (Axis-Shield). The cells from the interphase were washed three times with Dulbecco’s phosphate-buffered saline (DPBS; Invitrogen), counted and processed or cultured as described further.

### *Psoroptes ovis* antigen production

*Psoroptes ovis* mites of a heavily infested BB animal were collected, washed with DPBS and stored at −70 °C. For crude protein extract production, the mites were washed again with ice cold DPBS and crushed in liquid nitrogen in a pestle and mortar. This extract was centrifuged at 16 000 *g* for 30 min at 4 °C and sterilised over a 0.45 μm filter (Millipore). Using the Bradford method (Sigma-Aldrich), the concentration of the supernatant was determined at 10 mg/mL [30] after which the extract was stored at −70 °C.

### Histology, immunohistochemistry and cell counts

Tissue sections of the paraffin-embedded biopsies were stained with haematoxylin–eosin (HE) for the detection of eosinophils, toluidine blue staining for mast cells and two immunohistochemical stainings (CD3 staining for T-cells and CD20 staining for B-cells), based on Dreesen et al. [31]. In short, skin tissue sections of 4 μm were mounted on APES-coated slides, blocked with H_2_O_2_ and stained with polyclonal rabbit anti-human CD3 (Dako, Belgium) or rabbit anti-human CD20 (Sigma-Aldrich, USA) antibodies. T- and B-cells were visualized by adding peroxidase labelled goat anti-rabbit antibodies (Dako, Belgium), diaminobenzidine tetrahydrochloride (DAB; Dako, Belgium) and by performing a counterstaining with haematoxylin. Eosinophils, mast cells, T-cells and B-cells were quantified by taking two random pictures per tissue slide at 400× magnification on a *LEICA* light microscope and counting the positive cells on two tissue slides per animal. Results were expressed as the number of cells per 10^5^ μm^2^ tissue surface.

### Quantitative real-time polymerase chain reaction (qRT-PCR)

The frozen skin samples that were crushed in liquid nitrogen, homogenized and phase separated in TRIzol (Invitrogen) were used for RNA purification according to Grit et al. [32]. In short, RNA was extracted from the aqueous phase using the RNeasy kit (Qiagen). On-column DNase digestion was included by using the RNase-free DNase set (Qiagen) and RNA concentrations were measured with a Nano-Drop 2000 spectrophotometer. In addition, PBMC were re-stimulated with either DPBS or 5 μg/mL *P. ovis* antigen and cultured for 5 days in a 24-well flat-bottomed plate (BD Biosciences), after which the harvested cell pellets were washed 3 times with DPBS and stored at −70 °C or directly used for on-column RNA purification as described above. cDNA was generated from 100 to 150 ng total RNA using the iScript cDNA synthesis kit (Bio-rad). As the RNA yield from the PBMC of one uninfested HF was low, 60 ng RNA was used for the cDNA production of the PBMC from all uninfested HF (*n* = 4). The cDNA of all animals was diluted 1:10 in RNase free water and all qRT-PCR analyses were carried out as described by Dreesen et al. [33] using the SYBR Green Master Mix (Applied Biosystems) on 2 μL of single-stranded cDNA per reaction volume. All reactions were carried out in duplicate. Primer sequences and abbreviations for all genes that were tested are listed in Table 1. Using GeNorm software (geNorm 3.5), two breed specific internal control genes were selected from six candidate housekeeping genes: *GADPH*, *HPRT1*, *RLP0*, *SDHA*, *RPS29* and *HDAC10*. Analysis of the skin samples was performed with housekeeping genes *SDHA* and *RPS29* for BB and *HPRT1* and *SDHA* for HF. Additional genes were tested to evaluate skin related pathology (Table 1). New primer sets for three EDC genes; *filaggrin* (*FIL*), *involucrin* (*IVL*) and *loricrin* (*LOR*) were designed based on the following bovine reference gene sequences from the National Centre for Biotechnology Information (NCBI) database [34]: [*FIL*: XM_010826841, XM_010826842, XM_010826843], [*IVL*: XM_005203832, XM_010802998, XM_005203836] and [*LOR*: NM_001113757, XM_010826802, XM_010802876]. The sequences were aligned in SeqMan Pro (DNASTAR Lasergene) and primers were designed with Primer3web version 4.0.0 (free available online) (Table 1). Mean fold changes in gene transcription levels were obtained by comparing infested with control animals. For the analysis of the PBMC, housekeeping genes *GAPDH* and *HPRT1* were used for normalization in the BB animals and *RLP0* and *SDHA* for the HF. Relative quantities (Q values) were calculated using the delta Ct method to determine the fold differences in gene transcription levels of antigen re-stimulated cells of each animal compared to DPBS re-stimulated cells.

**Table 1:** Primer sequences (cattle) and abbreviations of the genes used in the qRT-PCR assays, including GeneBank accession numbers.

	Accession number	Primer sequence
Housekeeping genes		
*GAPDH* *(glyceraldehyde*-*3*-*phosphate dehydrogenase)*	NM_001034034.1	F: ACCCAGAAGACTGTGGATGG R: CAACAGACACGTTGGGAGTG
*HPRT1* *(hypoxanthine phosphoribosyltransferase 1)*	NM_001034035.1	F: CACTGGGAAGACAATGCAGA R: ACACTTCGAGGGGTCCTTTT
*RPS29* *(ribosomal protein S29)*	BC102702	F: GGAGCCATCCGAGAAAATTCG R: CAACTTAATGAAGCCGATGTCCTT
*RLP0* *(ribosomal protein P0)*	NM_001012682.1	F: CTTCATTGTGGGAGCAGACA R: GGCAACAGTTTCTCCAGAGC
*HDAC10* *(histone deacetylase 10)*	NM_001075460.1	F: CCGATGACGGGAGAAATCTA R: CTCAGGAACCCACCAGTTGT
*SDHA* *(succinate dehydrogenase complex subunit A)*	NM_174178.2	F: ACATGCAGAAGTCGATGCAG R: GGTCTCCACCAGGTCAGTGT
qRT-PCR PBMC and skin		
*IL*-*2* *(IL*–*interleukin)*	NM_180997.1	F: TCCAAGCAAAAACCTGAACC R: CAGCGTTTACTGTTGCATCATC
*IL*-*4*	NM_173921.2	F: GCGGACTTGACAGGAATCTC R: TCAGCGTACTTGTGCTCGTC
*IL*-*5*	NM_173922.1	F: TGGTGGCAGAGACCTTGACA R: TTCCCATCACCTATCAGCAGAGT
*IL*-*6*	NM_173923.2	F: TCCTTGCTGCTTTCACACTC R: CACCCCAGGCAGACTACTTC
*IL*-*10*	NM_174088.1	F: TGTATCCACTTGCCAACCAG R: CAGCAGAGACTGGGTCAACA
*IL*-*13*	NM_174089.1	F: GGTGGCCTCACCTCCCCAAG R: ATGACACTGCAGTTGGAGATGCTG
*IL*-*17*	NM_001008412.1	F: GGACTCTCCACCGCAATGAG R: TGGCCTCCCAGATCACAGA
*IL*-*23A*	NM_001205688.1	F: CCCGTATCCAGTGTGAGGAT R: AGTATGGAGGCGTGAAGCTG
*FOXP3* *(forkhead box P3)*	NM_001045933.1	F: GACAGCACCCTTTCGACTGT R: CTCCAGAGATTGCACCACCT
*IFN*-*γ* *(interferon*-*γ)*	NM_174086.1	F: TTCTTGAATGGCAGCTCTGA R: TTCTCTTCGGCTTTCTGAGG
*NCR1* *(natural cytotoxicity triggering receptor1)*	NM_183365.1	F: CTGAGAGCGTGGGTGTATCA R: CTGAGAGCGTGGGTGTATCA
*TGF*-*β1* *(transforming growth factor β1)*	NM_001166068.1	F: CTGCTGTGTTCGTCAGCTCT R: TCCAGGCTCCAGATGTAAGG
qRT-PCR skin		
*AREG* *(amphiregulin)*	BC141281.1	F: TGGTCA R: GTCGATCACGGAGGACAGTT
*CCR3* *(chemokine receptor 3)*	NM_001194960.1	F: TGTGTCAACCCCGTGATCTA R: AGAGTTCCTGCTCCCCTGTT
*FCERA1* *(Fc IgE receptor α)*	NM_001100310.1	F: CAGAGGCTGCCCTACATCTC R: GTTTAGGCTGTGGGTCCGTA
*FIL* *(filaggrin)*	*	F: GCCCAGTTCTAGACGCTGAC R: TCAAGCCAGTGACAGTGAGG
*IVL* *(involucrin)*	*	F: AAGGTCTTGGGCCAGCACTTG R: GATGCTGGGTTGTAACTCCCCCCAC
*LOR* *(loricrin)*	*	F: CAGTGGATCCGTCTGCCTGGGA R: CATGAGAGCGGTAAGCCCATCGAC

*Primers designed based on bovine reference gene sequences from NCBI database.

### Cell proliferation assays

#### ^3^H-thymidine uptake assay

For the ^3^H-thymidine uptake assay, 5 × 10^5^ cells per well were cultured in a 96-well round-bottomed plate (Thermo Scientific) using 200 μL of complete Roswell Park Memorial Institute (RPMI) cell culture medium, which consisted of RPMI-1640 (Invitrogen) supplemented with l-glutamine (Invitrogen), 10% foetal calf serum (Moregate), gentamycin (Invitrogen) and β-mercaptoethanol (Sigma-Aldrich). The cells were either re-stimulated with a negative control (DPBS), 5 μg/mL Concanavalin A (ConA, Sigma-Aldrich) as a positive control or *P. ovis* crude protein antigen at 5, 10, 25 or 50 μg/mL. All conditions were performed in triplicate. The cells were pulsed with 1 μCi ^3^H-thymidine (Perkin Elmer) after 4 days of culture. After 18 h of incubation, the cells were harvested and the incorporated radioactivity was measured using a β-scintillation counter (Perkin Elmer). Results are shown as stimulation indices (SI), which are the ratios of the counts per minute of *P. ovis* re-stimulated cells and the counts per minute of the negative control.

#### PKH staining and flow cytometry

To identify which cell populations from the whole PBMC fraction were proliferating after antigen re-stimulation, isolated PBMC were labelled with PKH using the PKH26 red fluorescent cell linker mini kit (Sigma-Aldrich) according to the manufacturer’s protocol. A small fraction of the stained cells was used to determine the PKH starting intensity by flow cytometry analysis. The rest of the labelled cells was cultured at 5 × 10^5^ cells per well in a 96-well round-bottomed plate (Thermo Scientific) in 200 μL complete RPMI cell culture medium and re-stimulated with DPBS or 5 μg/mL *P. ovis*. All conditions were performed in duplicate.

After 5 days of culture, the cells were harvested and two cell stainings were performed in DPBS supplemented with 1% bovine serum albumin (BSA, Sigma-Aldrich) and 0.1% Na-azide (Sigma-Aldrich) by using the following monoclonal primary antibodies: CD3 (mouse IgG1, clone MM1A, VMRD), TCRγδ (mouse IgG2b, clone GB21A, VMRD), CD4 (mouse IgG2a, clone CC8) and CD8 (mouse IgM, clone BAQ111A, VMRD) in the first staining. CD3, CD21 (mouse IgM, clone BAQ15A, VMRD) and Alexa Fluor 488-labeled CD335 (mouse IgG2b, clone AKS6) in the second staining. The CD335 antibody was kindly provided by Prof. A. Storset (School of Veterinary Medicine, Norway). Bound mAb were detected using the following secondary antibodies: rat anti-mouse IgG1-V450 (BD Biosciences), rat anti-mouse IgG1-APC (BD Biosciences), rat anti-mouse IgG2b-FITC (Southern Biotech), goat anti-mouse IgG2a-APC (Invitrogen) and rat anti-mouse IgM-APCCy7 (Biolegend). The PKH intensity for each cell population was determined on a FACSAriaIII (BD Biosciences). Viable cells were gated based on forward and side scatter and lack of propidium iodide (Molecular Probes) uptake. Doublets were eliminated from the analysis by gating on forward scatter-height and forward scatter-area. T-cells were identified as CD3+ cells, T-helper cells as CD3+/CD4+ and cytotoxic T-cells as CD3+/CD8+. In the CD3− population, CD21+ cells were classified as B-cells, CD335+ cells as natural killer (NK)-cells and a third population of bovine CD3−/CD21−/CD335− cells was clearly defined but could not be identified so far.

Data were analysed using FlowJo software (Tree Star) and quantified using ModFit LT software (Verity Software House). Proliferation indices (PI) were calculated for each separate cell population and compared between DPBS and *P. ovis* antigen re-stimulated cells.

### Statistical analysis

GraphPad Prism was used to perform all statistical analyses. For the comparison of the stimulation indices and Q values (qRT-PCR on skin biopsies) between infested and uninfested animals, a nonparametric Mann–Whitney U test was performed. A one-sided test was used for the stimulation indices and a two-sided test for the Q values. Proliferation indices generated from the PKH staining and Q values of unstimulated vs. re-stimulated cells (qRT-PCR on PBMC) were compared using a nonparametric Wilcoxon signed-rank test: one-sided for the PKH data and two-sided for the Q values. *P* values ≤0.05 were considered statistically significant.

## Results

### In vivo cutaneous immune response

Results of the eosinophil, T- and B-cell and mast cell counts in the skin of infested and uninfested BB and HF animals are listed in Table 2. The number of eosinophils (46.7 per 10^5^ μm^2^), T-cells (83.8 per 10^5^ μm^2^) and B-cells (30.6 per 10^5^ μm^2^) was significantly higher in the infested BB than in the control animals (6.5 per 10^5^ μm^2^, 33.8 per 10^5^ μm^2^ and 5.9 per 10^5^ μm^2^ respectively), whereas the number of mast cells did not significantly differ. In HF animals, eosinophil, T-cell, B-cell and mast cell counts in the skin appeared to be higher in infested animals compared to the uninfested controls, although cell counts were not significantly different between the groups (Table 2).

**Table 2:** Histological cell counts in skin biopsies of control animals and *Psoroptes ovis* infested cattle.

	T-cells (CD3+)	B-cells (CD20+)	Eosinophils	Mast cells
A				
Control (*n* = 8)	33.8 ± 4	5.9 ± 1.1	6.5 ± 1.3	12.4 ± 1.2
Infested (*n* = 12)	83.8 ± 7.8*	30.6 ± 6.7*	46.7 ± 4.9*	14.3 ± 2.6
B				
Control (*n* = 4)	34.5 ± 11.7	5.8 ± 0.8	6.4 ± 2.8	12.5 ± 1.2
Infested (*n* = 4)	46.6 ± 5.4	10.6 ± 4	35.1 ± 10	19 ± 4.6

Panel A: Cell counts from Belgian Blue cattle. Panel B: Cell counts from Holstein–Friesian cattle. Data are presented as mean number of cells per 10^5^ μm^2^ ± SEM (* *P* < 0.05).

**Figure 1:**
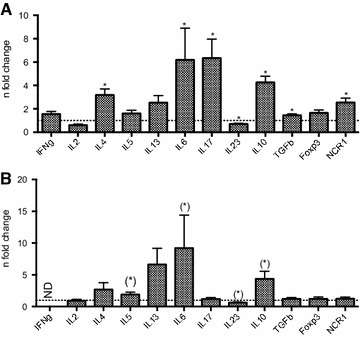
**Gene transcription profile in the skin of Psoroptes ovis infested Belgian Blue (A) and Holstein–Friesian cattle (B)**. qRT-PCR data is presented as mean fold changes in infested animals (BB *n* = 11; HF *n* = 4) compared to uninfested controls (BB *n* = 8; HF n = 4; dashed horizontal line) with SEM as error bars. *P < 0.05, (*) *P* = 0.057.

To identify whether specific cytokines characteristic for a Th1, Th2 or Th17 immune response are produced during infection, qRT-PCR was performed on skin biopsies of uninfested and infested BB and HF animals (Figure 1). Only 11 infested BB animals were included as from one animal an insufficient amount of RNA was obtained. A mixed Th2/Th17 cytokine profile was observed in the skin of infested BB with an up-regulation of IL-6 (6.2-fold; *P* = 0.043) and IL-17 (6.3-fold; *P* = 0.006), as well as up-regulated transcription of IL-4 (3.2-fold; *P* = 0.012), IL-5 (1.6-fold; *P* = 0.149), IL-13 (2.5-fold; *P* = 0.107) and IL-10 (4.3-fold; *P* = 0.001). TGF-β and NRC1 were also up-regulated in infested animals (1.4- and 2.5-fold and *P* values of 0.006 and 0.003 respectively). Cutaneous transcription of IL-23 was down-regulated (0.7-fold; *P* = 0.019) and a low IFN-γ (1.6-fold; *P* = 0.159) response was observed.

In general, these responses were comparable to those in HF cattle. The cytokine profile in the skin of HF demonstrated a predominant Th2-like response with up-regulated transcription of IL-4 (2.7-fold; *P* = 0.686), IL-5 (1.9-fold; *P* = 0.057), IL-13 (6.6-fold; *P* = 0.124) and IL-10 (4.4-fold; *P* = 0.057). Virtually no change in IL-17 transcription (1.2-fold; *P* = 0.343) was observed, while IL-6 was up-regulated (9.2-fold; *P* = 0.057). No significant changes in IL-23 transcription were observed in the skin (0.6-fold; *P* = 0.057). IFN-γ was up-regulated in three out of four infested animals, but could not be quantified as the transcription in the uninfested control animals was too low to detect.

### In vitro immune response after PBMC re-stimulation

**Figure 2:**
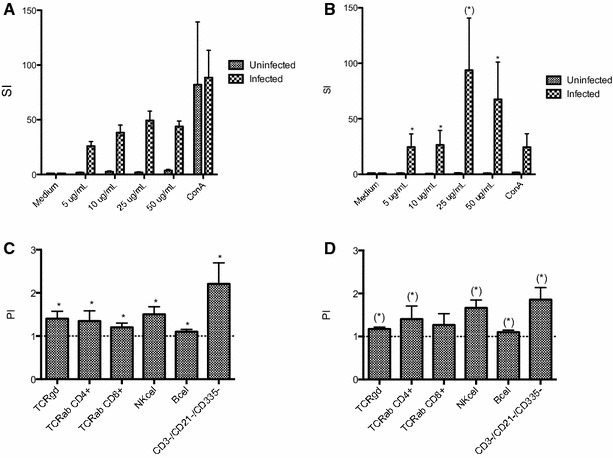
^**3**^ **H-thymidine uptake assay and PKH staining of circulating PBMC from (un)infested Belgian Blue and Holstein–Friesian cattle.** **A** Mean stimulation index (SI ± SEM) of PBMC from uninfested (n = 8) and infested (n = 12) BB cattle re-stimulated with medium, 4 different concentrations of *P. ovis* and ConA. **B** Mean stimulation index (SI ± SEM) of PBMC from uninfested (*n* = 4) and infested (*n* = 4) HF cattle re-stimulated with medium, four different concentrations of *P. ovis* and ConA. **C** Mean proliferation index (PI ± SEM) of PKH labelled PBMC re-stimulated with 5 μg/mL *P. ovis* antigen compared to unstimulated PBMC (dashed horizontal line) of infested BB (*n* = 12). **D** Mean proliferation index (PI ± SEM) of PKH labelled PBMC re-stimulated with 5 μg/mL *P. ovis* antigen compared to unstimulated PBMC (dashed horizontal line) of infested HF (*n* = 4). *P < 0.05, (*) *P* = 0.057.

Proliferation of re-stimulated PBMC was assessed using a ^3^H-thymidine uptake assay, in which cells from uninfested and infested BB and HF animals were re-stimulated with either DPBS as negative control, ConA as positive control or several concentrations of *P. ovis* antigen (5, 10, 25 or 50 μg/mL). Results demonstrated a significant and largely concentration-dependent PBMC proliferation in infested BB and HF compared to the uninfested animals (Figures 2A and B).

Using PKH staining on cells of infested BB and HF re-stimulated with 5 μg/mL antigen or DPBS, a significant antigen specific proliferation in all investigated cell populations (αβ T-cells, γδ T-cells, B-cells, NK-cells and CD3−/CD21−/CD335− cells) was observed, with the highest reaction seen in NK- and CD3−/CD21−/CD335− cells (Figures 2C and D).

**Figure 3:**
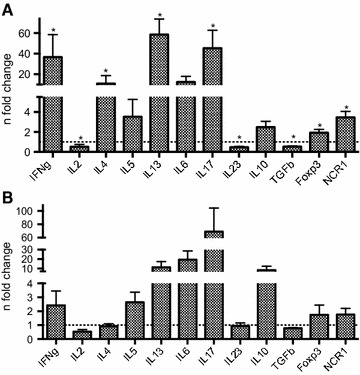
**qRT-PCR results of circulating PBMC from infested Belgian Blue and Holstein–Friesian cattle.** **A** Gene transcription profile in PBMC of infested BB (*n* = 11). **B** Gene transcription profile in PBMC of infested HF (*n* = 4). qRT-PCR results of PBMC re-stimulated with 5 μg/mL *P. ovis* antigen are presented as mean fold changes in re-stimulated compared to unstimulated cells (dashed horizontal line) (*P < 0.05).

It was subsequently investigated whether circulating re-stimulated PBMC of infested BB and HF animals after in vitro re-stimulation with *P. ovis* antigen produced similar cytokines as in the skin (Figure 3). As the RNA yield of one infested BB animal was insufficient to perform qRT-PCR, data of 11 instead of 12 BB animals is shown in Figure 3A. In parallel to the cutaneous immune response, re-stimulation of PBMC in infested BB induced a predominant Th2- and Th17-like response with up-regulated transcription of IL-4 (11-fold; *P* = 0.019), IL-13 (58.6-fold; *P* = 0.001) and IL17 (45.5-fold; *P* = 0.003). In addition, an up-regulation of Foxp3 (1.9-fold; *P* = 0.032) and NCR1 (3.5-fold; *P* = 0.003) transcription was observed, as well as of IFN-γ (36.8-fold; *P* = 0.003), IL-5 (3.5-fold; *P* = 0.413), IL-10 (2.5-fold; *P* = 0.102) and IL-6 (12.5-fold; *P* = 0.831). TGF-β, IL-2 and IL-23 transcription were down-regulated (all 0.5-fold with *P*-values of 0.001, 0.005 and 0.001 respectively).

The cytokine profile of circulating re-stimulated PBMC in infested HF animals (Figure 3B) revealed a mixed Th2/Th17-like response with 2.6-, 11.5- and 19.4-fold changes for IL-5, IL-13 and IL-6 respectively. IL-17 and IL-10 were consistently up-regulated in all samples (average fold changes of 69.1 and 8.5 respectively), systemic IFN-γ was 2.4-fold up-regulated and IL-23, TGF-β and IL-2 were slightly down-regulated, with 0.9-, 0.8- and 0.5-fold changes respectively. However, the statistical significance for these data could not be interpreted due to the combination of a low number of animals (*n* = 4) and the use of a two-sided paired statistical test.

**Figure 4:**
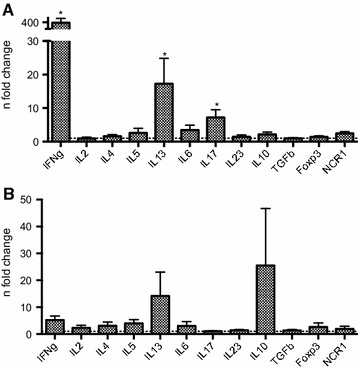
**qRT-PCR results of circulating PBMC from uninfested Belgian Blue and Holstein–Friesian cattle.** **A** Gene transcription profile in PBMC of uninfested BB (*n* = 8). **B** Gene transcription profile in PBMC of uninfested HF (*n* = 4). qRT-PCR results of PBMC re-stimulated with 5 μg/mL *P. ovis* antigen are presented as mean fold changes in re-stimulated compared to unstimulated cells (dashed horizontal line) (*P < 0.05)

In order to evaluate whether these observations were mainly due to a cellular memory response or whether innate reactions were also responsible, the same procedure was followed for the uninfested BB (*n* = 8) and HF (*n* = 4) animals with the results listed in Figure 4. A 17.3-fold (*P* = 0.039), 7.2-fold (*P* = 0.031) and 376.8-fold (*P* = 0.039) up-regulation of respectively IL-13, IL-17 and IFN-γ was observed in BB animals (Figure 4A). Finally, qRT-PCR results from the re-stimulated PBMC of uninfested HF animals are listed in Figure 4B and show an up-regulation of IL-13 (14.2-fold) and IL-10 (25.5-fold). In contrast with the PBMC from uninfested BB cattle, virtually no IL-17 response and a moderate IFN-γ up-regulation (1.9- and 5.3-fold respectively) were observed in uninfested HF. For the results in HF, the statistical significance could not be interpreted for the same reasons as mentioned above.

## Discussion

In this study, the cutaneous immune response and in vitro cellular immune reaction in BB and HF cattle during natural *P. ovis* infestation were analysed in order to identify a potential cause of the high susceptibility of the BB breed to this parasitic infestation. A significant influx of immune cells in the skin of infested BB was observed, coinciding with a mixed Th2/Th17 cytokine profile. A largely similar cytokine pattern could be elicited when circulating PBMC from infested BB were re-stimulated with *P. ovis* antigen in vitro. Moreover, a significant and largely antigen concentration-dependent PBMC proliferation was observed in infested animals compared to uninfested controls, with proliferation of all investigated cell subpopulations (αβ T-cells, γδ T-cells, B-cells, NK-cells and CD3−/CD21−/CD335− cells). This demonstrates the presence of antigen specific memory T-cells, and it confirms previous assumptions that an impaired cellular immune response in BB is not the cause of the high susceptibility of this breed [5]. HF animals are known to display less severe symptoms during *P. ovis* infection in comparison to BB, which is reflected in the lower CI of the animals in this study and the less pronounced influx of immune cells in infested skin. Although the cytokine pattern observed in the skin was similar to that in BB, no cutaneous IL-17 production was observed in infested HF. Furthermore, circulating PBMC from infested HF also produced a less pronounced Th2-like response. It should however be stressed that a different mite exposure in vivo could be responsible for these observations. Furthermore, the infestation stage of the animals was unclear and there was a small age difference between the animals, which could also partly explain the differences between both breeds.

Th2 and Th17 cytokines have been described as being important in parasitic skin diseases. Production of Th2 cytokines is often observed in ectoparasite infections in cattle as it is in dogs, rabbits and mice with scabies [17, 18, 26, 27]. The cutaneous mixed Th2/Th17 profile that is observed in BB could potentially be associated with more severe symptoms, as described in pigs with aggravating symptoms of *Sarcoptes scabiei* and in allergic diseases in humans [23, 28, 35]. Although this specific immune response could merely be a reaction to the presence of severe symptoms linked with high numbers of mites, the reversed hypothesis is also plausible. Indeed, Th2 cytokines produced in the skin will not only stimulate local immune cells to attack the mites, but will also cause collateral damage to the surrounding tissue, leading to the clinical symptoms. Despite the IL-23 down-regulation, the additional pro-inflammatory Th17-like response in BB could intensify these effects leading to a more severe clinical phenotype. As mite counts were not performed in this study, it remains unsure whether this Th2/Th17-like response in infested BB animals is either not protective or is protective but “uncontrollable”. Little to no signs of a Treg reaction that could dampen this pro-inflammatory response, were noticed in both breeds, as only IL-10, which is also a Th2 cytokine, was up-regulated, without transcription of TGF-β. Furthermore, only a diminutive up-regulation of Foxp3, a transcription factor that is generally expressed by Treg T-cells [36, 37] was noticed. Remarkably, little to no IFN-γ was transcribed in the skin of infested BB, while substantial up-regulation of this pro-inflammatory cytokine was observed in re-stimulated PBMC from infested and uninfested BB. The latter indicates that IFN-γ could mainly be released by innate immune cells, such as macrophages, NKT- or NK-cells [36]. Low levels of this cytokine in the skin could be caused by the fact that the adaptive immune response already took over at this point of infection. Production of IFN-γ, a pro-inflammatory cytokine that pushes naïve T-cells towards Th1-cells [36], has been described in murine spleen and lymph node cells in vitro after challenge infection with *Sarcoptes scabiei* [17]. In contrast with the uninfested BB, low IFN-γ and high IL-10 levels were observed in the re-stimulated PBMC from uninfested HF animals. It remains unclear why IFN-γ and IL-10 transcription in HF does not resemble the pattern observed in BB and it should be further investigated whether this could be part of the cause of different breed susceptibility.

The mixed cytokine profile seen in BB may have an effect on several immune cells downstream. Th2 cytokines are known to promote the immune response to parasites: IL-4 and IL-13 play a role in the induction of allergy as they stimulate B-cell growth and IgE production, whilst IL-5 typically induces eosinophil differentiation and growth [36]. The pro-inflammatory effect of IL-17 causes recruitment of neutrophils and further promotes the attraction of eosinophils in the skin [35, 38]. Indeed, besides the recruitment of T- and B-cells, cell counts demonstrated a distinct influx of eosinophils in the skin of affected BB and a greater eosinophil skin infiltration has also been documented in sheep breeds susceptible to *P. ovis* and cattle breeds susceptible to tick infestations [11, 22]. The presence of high levels of IL-17 in the skin has also been linked to delayed-type hypersensitivity in humans [38], which could explain the distinctive intradermal skin test response observed by Losson et al. [5, 7]. In this study, no suppression of the transcription of EDC genes could be observed (results not shown), unlike data available in sheep. As a down-regulation of these genes in sheep skin is seen within 24 h after infection, it is possible that this was missed in this study [9, 13].

In summary, BB cattle display a mixed Th2/Th17 immune pathway during natural infection with *P. ovis*. We hypothesize that, in line with results from scabies affected humans and pigs, this might explain part of their susceptibility since no cutaneous Th17 profile could be detected in the more resistant HF breed. Furthermore, in contrast to the results from the HF, high transcription levels of IFN-γ and low IL-10 transcription in the uninfested and infested BB could indicate a potential role for these cytokines in the innate immune reaction against the mite. These differences in IFN-γ and IL-10 transcription could therefore also be partly responsible for the observed difference in breed susceptibility to mange. Further research is needed to identify potential cell sources and biological functions for these up-regulated cytokines and to fully unravel the basis of this different breed susceptibility to *P. ovis*.
